# Prediction of Protein Secondary Structures Based on Substructural Descriptors of Molecular Fragments

**DOI:** 10.3390/ijms252312525

**Published:** 2024-11-21

**Authors:** Oleg S. Zakharov, Anastasia V. Rudik, Dmitry A. Filimonov, Alexey A. Lagunin

**Affiliations:** 1 Department of Bioinformatics, Pirogov Russian National Research Medical University, 117997 Moscow, Russia; 2 Department of Bioinformatics, Institute of Biomedical Chemistry, 119121 Moscow, Russia

**Keywords:** prediction of secondary structures of protein, MNA descriptors, sequence–structure–property relationships, MultiPASS

## Abstract

The accurate prediction of secondary structures of proteins (SSPs) is a critical challenge in molecular biology and structural bioinformatics. Despite recent advancements, this task remains complex and demands further exploration. This study presents a novel approach to SSP prediction using atom-centric substructural multilevel neighborhoods of atoms (MNA) descriptors for protein molecular fragments. A dataset comprising over 335,000 SSPs, annotated by the Dictionary of Secondary Structure in Proteins (DSSP) software from 37,000 proteins, was constructed from Protein Data Bank (PDB) records with a resolution of 2 Å or better. Protein fragments were converted into structural formulae using the RDKit Python package and stored in SD files using the MOL V3000 format. Classification sequence–structure–property relationships (SSPR) models were developed with varying levels of MNA descriptors and a Bayesian algorithm implemented in MultiPASS software. The average prediction accuracy (AUC) for eight SSP types, calculated via leave-one-out cross-validation, was 0.902. For independent test sets (ASTRAL and CB513 datasets), the best SSPR models achieved AUC, Q3, and Q8 values of 0.860, 77.32%, 70.92% and 0.889, 78.78%, 74.74%, respectively. Based on the created models, a freely available web application MNA-PSS-Pred was developed.

## 1. Introduction

Secondary structures of proteins (SSPs) are specific, conformationally stable regular arrangements of amino acid chains formed by hydrogen bonding between the backbone atoms of the polypeptide chain. These structures are mainly categorized into α-helices, β-sheets, and the turns and loops that connect them. The precise folding and arrangement of amino acids into SSPs is critical for the biological activity of a protein, including enzymatic catalysis, signal transduction, and molecular recognition.

Predictions of SSPs are used in many areas of fundamental and applied studies in biology and medicine, including the development of drugs, vaccines, and therapeutic methods. For example, in pharmacology, antibodies such as rituximab use β-sheets in their variable regions to achieve high specificity in targeting cancer cells, demonstrating the importance of knowledge of secondary structures of proteins in the design of effective therapeutics [1,2]. The changing of SSPs due to amino acid substitutions may be a cause of hereditary diseases, and the determination of SSP disruptions is very important for the diagnosis of diseases by medical geneticists [3].

The Protein Data Bank (PDB) includes information on hundreds of thousands experimentally determined 3D structures of proteins [4]. These data can be used as a source of known SSPs using the Dictionary of Secondary Structure in Proteins (DSSP) software for their annotation [5]. The special accuracy metrics Q3 and Q8 are used to estimate the accuracy of computational methods for the prediction of SSPs. Q3 is a metric that evaluates the accuracy of predicting three common SSPs: helix (H), strand (E), and coil (C) [6]. Q8 is a metric that evaluates the accuracy of predicting eight SSPs: α-helix (H), 3_10_ helix (G), π-helix (I), polyproline II-helix (P), β-strand (E), unstructured coil (C), turn (T), and bend (S) [6] (the appropriate equations for the calculation of Q3 and Q8 are presented in Material and Methods).

One of the earliest and well-known methods for predicting SSPs is the GOR method [7]. This approach utilizes information theory and the Bayesian algorithm to establish relationships between the sequence of amino acids and the probability of forming specific secondary structures. The GOR method employs a sliding window of 17 amino acid residues to predict the structure of the central residue, incorporating information from eight residues on either side. The method predicts four types of secondary structures: α-helices (H), β-strands (E), coils (C), and turns (T). The GOR method was trained on 267 protein sequences, and its predictions are based on the frequencies of observed residue conformations within the sliding window. The training set used in the GOR method consisted of protein sequences longer than 50 residues with a resolution better than 2.5 Å and R-factor (the agreement between the experimental diffraction data and simulated experimental data calculated based on the 3D model of the biomolecule; see PDB documentation) less than 25%. The last version of GOR, GOR V, was trained on secondary structures from 513 proteins and displayed a Q3 accuracy, calculated by a leave-one-out cross-validation procedure, equal to 73.5% [8]. Although it has been a fundamental tool in comprehending SSPs, its limitations emphasize the necessity for more accurate approaches.

The modern state-of-the-art models to predict SSPs utilize a combination of advanced deep learning techniques to capture complex relationships within protein sequences. 

The secondary structure recurrent encoder-decoder networks (SSREDNs) model is a deep recurrent network featuring feed-forward layers and recurrent architectures [9]. It consists of an encoder part, a decoder part, and a representation layer. The encoder, using GRUs, captures both immediate and long-term amino acid dependencies, while the decoder translates these representations into predictions of SSPs. The SSREDNs model has been trained on datasets like CullPDB and CB513, achieving Q8 accuracies of 68.2% and 73.1% on CullPDB and CB513, respectively, outperforming many existing models. The key feature of this algorithm is its ability to leverage bidirectional GRUs to capture context-dependent relationships among residues, enhancing the accuracy of SSP prediction.

DeepACLSTM employs asymmetric convolutional operations and bidirectional LSTM (BLSTM) to predict SSPs [10]. The model’s asymmetric convolution filters and BLSTM layers efficiently extract local contexts and long-distance dependencies within protein sequences. The training involves normalization of input sequences to a fixed length and the application of dropout and early-stopping techniques to prevent overfitting. DeepACLSTM achieved a Q8 accuracy of 70.5% on the CB513 dataset, highlighting its effectiveness in capturing complex sequence–structure relationships. The model is trained using adaptive moment estimation (Adam) and is implemented using the Keras API with TensorFlow backend, providing robust performance across various datasets.

The CRRNN model integrates a local block, three stacked bidirectional GRU layers, residual connections, and fully connected layers to predict SSPs [6]. The local block uses 1D CNN to capture local sequence features, while the BGRU layers handle long-range interactions. The model is optimized using the Adam optimizer and trained with dropout to mitigate overfitting. CRRNN achieved Q8 accuracy of 71.4% on the CB513 dataset, demonstrating competitive performance with state-of-the-art methods. It effectively combines local and global sequence features, making it a powerful tool for SSP prediction.

The WGACSTCN model combines wide-gated attention mechanisms and temporal convolutional networks (TCNs) to capture both local and long-range dependencies in protein sequences [11]. It uses one-hot encoding and PSSM for feature representation and evaluates performance using Q3 and Q8 accuracies. The model achieved Q3 and Q8 accuracy of 84.97% and 75.7%, respectively, on the CB513 dataset, showcasing its superior ability to predict SSPs. WGACSTCN’s architecture includes WGAN-GP modules for feature extraction and CBAM-TCN residual blocks to enhance long-range interaction modeling, resulting in improved accuracy and robustness.

AttSec employs a transformer-based architecture to predict SSPs by capturing hierarchical properties and local patterns [12]. The model uses relative position encoding to consider the importance of amino acid distances in hydrogen bond formation. AttSec achieved Q8 accuracy of 75.2% on the CB513 dataset, outperforming many models by leveraging deep attention mechanisms and convolutional blocks to accurately predict secondary structures. The model’s design allows it to effectively utilize protein embeddings and long-range dependencies, providing the state-of-the-art performance across multiple datasets.

Several web applications for SSP prediction have also existed and been supported for many years. PCIPRED 4.0 is based on deep neural networks and position-specific scoring matrices generated by PSI-BLAST (http://bioinf.cs.ucl.ac.uk/psipred/) [13]. JPred4 is based on a neural network, multiple alignment made by PSI-BLAST, and hidden Markov models (https://www.compbio.dundee.ac.uk/jpred4/) [14]. COUDES was based on PSIPRED prediction and improved it by using propensities weighted by scores coming from position-specific scoring matrices (PSSMs) generated by PSI-BLAST (https://bioserv.rpbs.univ-paris-diderot.fr/services/COUDES/) [15]. According to the appropriate publications, they provide reasonable accuracy of prediction on test sets (close to the above-mentioned methods).

The estimation of secondary structure of proteins may also be given from the results of AlphaFold prediction, which has revolutionized protein structure prediction by accurately modeling 3D structures of proteins from amino acid sequences [16]. AlphaFold leverages evolutionary data, multiple sequence alignments, and structural templates to predict the overall 3D fold of proteins, including secondary structure elements, but this is done within the context of full structure prediction. Therefore, it is not directly comparable to the methods focused on specific secondary structure predictions evaluated in our study, which primarily target sequence-based predictions rather than holistic structural modeling.

Current approaches primarily leverage sequence-based methods and exploit evolutionary information to predict SSPs using machine learning models that identify patterns in amino acid sequences correlated with specific structural motifs. These models use features such as residue interactions, physicochemical properties, and evolutionary profiles from multiple sequence alignments to infer probable folding patterns, capturing intricate dependencies and long-range interactions. Current machine learning models are unable to accurately predict SSP due to the inherent complexity and variability of protein folding, which involves numerous factors that are challenging to comprehensively capture. While they are promising, these models’ predictions require further refinement to achieve higher accuracy and reliability.

In this study, we propose a new sequence–structure–property relationships (SSPR) modeling approach to create classification SSPR models for the prediction of SSPs based on representation of their sequences as structural formulae. This approach allows for a nuanced representation of molecular structure, capturing essential chemical characteristics without relying on specific bond configurations. SSPR modeling has been implemented in MultiPASS software and successfully used for the creation of SSPR models predicting the pathogenicity [17,18,19] and drug resistance [20] of amino acid substitutions, epitopes, and MHC specificity for CDR3 TCR sequences [21] and phosphorylation sites [22].

## 2. Results

**Figure 1:**
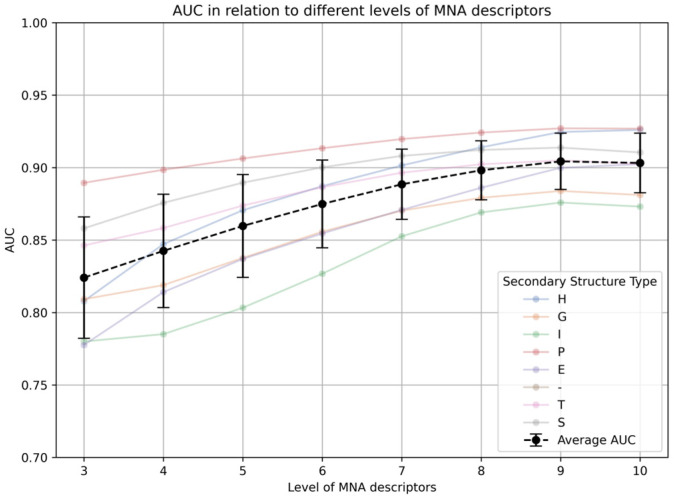
AUC relative to different levels of MNA descriptors.

MultiPASS uses different levels of MNA descriptors. We studied how the levels of MNA descriptors relate with the accuracy of SSPR models constructed on the basis of the created training set in the SD file. The study was evaluated by the calculation of the accuracy of predictions (AUC, the area under the curve) using the leave-one-out cross-validation (LOO CV) procedure for each SSPR model related with the appropriate level of MNA descriptors (Figure 1).

Increase in the level of MNA descriptors improves the accuracy of predictions (AUC) for various secondary structure types up to the 9th level of MNA descriptors. Therefore, the model created on this level of MNA descriptors was chosen as optimal. The accuracy of prediction (AUC_LOO CV_) and the number of positive records (N) for each type of SSP are represented in Table 1.

The performance of the best SSPR model in predicting SSPs was assessed in comparison with other methods using several metrics, including sensitivity, specificity, accuracy, balanced accuracy, and AUC. These metrics are calculated for different types of SSP across two datasets, ASTRAL and CB513. Two new training sets were created excluding from the initial training set all data from ASTRAL and CB513, respectively. These training sets were used to create two new SSPR models based on the 9th level of MNA descriptors, which were used for the prediction of SSP for the ASTRAL and CB513 test sets. The accuracy of prediction (AUC_LOO CV_) and number of positive records (N) for each type of SSP for new SSPR models are represented in Table 1 (see columns “Model without ASTRAL data” and “Model without CB513 data”).

Table 1 shows that after excluding the data of the ASTRAL and CB513 test sets from the initial training set, the accuracy of SSPR models estimated by the LOO CV procedure remained approximately the same. We used these SSPR models for the prediction of SSP types for the ASTRAL and CB513 test sets, which are considered as independent external test sets. The accuracy metrics for the given results at P_a_ > P_i_ as a threshold are represented in Table 2.

For the ASTRAL test set, the average balanced accuracy and AUC values are 0.784 and 0.860, while for the CB513 dataset, they are slightly higher at 0.817 and 0.889, respectively. Balanced accuracy is a useful metric in this context because it accounts for imbalances in the dataset by averaging the true positive rate (sensitivity) and the true negative rate (specificity). This ensures that the model’s performance is evaluated fairly, even when some classes are underrepresented.

In examining the class-specific metrics for the ASTRAL test set, we see that the H class has a high sensitivity of 0.823 and specificity of 0.840, resulting in a balanced accuracy of 0.832. The AUC value for the H class is also highest, at 0.907. This indicates that the model is particularly adept at predicting α-helices, likely due to their distinctive and regular hydrogen-bonding patterns, which are easier to capture using MNA descriptors. However, classes G, I, and T show much lower sensitivities (0.766, 0.612, and 0.782, respectively), contributing to their lower balanced accuracies (0.772, 0.693, and 0.796). These structures are less regular and more context-dependent, making them harder to predict accurately. The prediction for the C class is also less accurate owing to the more diverse structures of sequences.

For the CB513 dataset, the H class again shows a high value of balanced accuracy (0.852), reflecting consistent performance in predicting α-helices across datasets. The E class also exhibits good performance, with a balanced accuracy of 0.823, due to its relatively straightforward β-sheet structure that the model can capture well. However, classes G, I, and C also show the lowest balanced accuracies (0.790, 0.765, and 0.774, respectively). These low values highlight the model’s difficulty in predicting irregular or less common secondary structures.

The analysis of the prediction results in Table 2 shows that there are a significant number of false positive predictions for G (3_10_-helix), I (π-helix), P (polyproline II-helix), T (turn) and C (unstructured coil). This may be due to the fact that a significant part of the G, P, T, and C sequences in the training set also belong to other types of SSP (see the table in "Material and Methods). Many of the overpredicted SSP sequences belonging to the G, P, and T types are short or include overrepresented amino acid residues or short repeats. For example, the prediction results for FGAVGAL (types E and H in the PDB entries) include positive predictions for SSP types I, H, T, and G. The prediction results for DGGLLL (types S and T according to the PDB entries) include SSP types S, T, G, and C. The prediction results for RNQR (types P and C in the PDB) include SSP types T, G, P, S, and C.

For the ASTRAL test set, the SSPR model achieved a Q8 accuracy of 70.92% and Q3 accuracy of 77.32%, showcasing its ability to predict secondary structure elements with a reasonable accuracy despite excluding 25% of SSP structures from the initial training set. On the CB513 test set, the SSPR model achieved a Q8 accuracy of 74.74% and Q3 accuracy of 78.78%, indicating its robustness in predicting SSP across different datasets. When compared to other models on the CB513 dataset, the SSPR model’s Q3 and Q8 accuracies of 78.78% and 74.74% are competitive, although it falls short of some advanced models. For instance, the SSREDN model reported a Q3 accuracy of 82.9% (Q8—73.1%), the CRRNN model achieved Q3 accuracy of 82.9% (Q8—71.4%), the WGACSTCN model reported 84.97% (Q8—75.7%), and the AttSec model reached 86.5% (Q8—75.2%). The performance of the SSPR model, evaluated using the Q8 and Q3 accuracy metrics, demonstrates its competitive advantages in predicting SSP. Despite these comparisons, the SSPR model’s performance remains noteworthy and highlights areas for further improvement.

**Figure 2:**
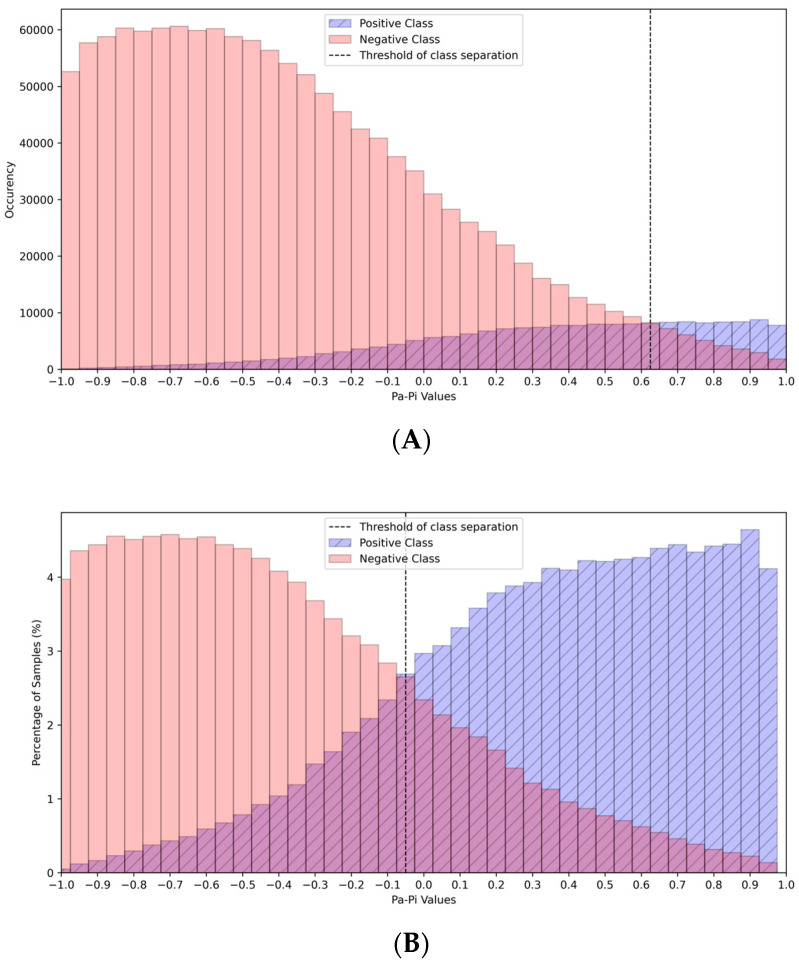
The plots of dependence between P_a_-P_i_ values and absolute (**A**) and % (**B**) numbers of predicted positive and negative cases created on the prediction results for the data from the ASTRAL and CB513 test sets.

To help in interpreting the predicted results, we created plots with the absolute (Figure 2A) and % (Figure 2B) numbers of predicted positive and negative cases depending on P_a_-P_i_ values based on the prediction results for the data from the ASTRAL and CB513 test sets. These plots were generated by first calculating the distribution of P_a_-P_i_ values for both positive and negative classes. In the absolute occurrence plot (Figure 2A), histograms for each class were constructed using 40 bins ranging from -1 to 1, and the absolute number of occurrences in each bin was plotted. The threshold for class separation was marked at the P_a_-P_i_ value of 0.625. In the percentage plot (Figure 2B), the histogram counts were converted to percentages, showing the relative distribution of P_a_-P_i_ values within each class. Here, the threshold for class separation was set at −0.05.

The data in Figure 2 confirm that as P_a_-P_i_ values increase, the proportion of true positive predictions also increases, and the proportion of false positive predictions decreases, as some positives may be estimated by a P_a_-P_i_ value less 0.

We have implemented the best SSPR models in the MNA-PSS-Pred (MNA-based Protein Secondary Structure Predictor) web application, which is freely accessible at https://www.way2drug.com/MNA2DFinder/. MNA-PSS-Pred has a user-friendly interface where everyone can input UniProt ID or protein sequences in the FASTA format in the query box and obtain the predicted SSPs along with their likelihood scores. MNA-PSS-Pred also provides a mode to predict SSPs for up to 100 peptides at once.

The web application in the Protein mode processes the protein sequence, generating predictions for all possible subsequences converted in an SD file by sliding the window ranging from 4 to 30 amino acids in length. Each position of a residue in a protein was annotated by the maximal predicted P_a_-P_i_ value among all subsequences. In the peptide mode, a query sequence(s) was converted to its structural formula(e) for prediction. The results display the predicted secondary structures, visualized in a color-coded format, allowing users to easily identify different types of structures, such as eight (SSP (all types)) or three types of classification (SSP (three types)), including helices, strands, and unstructured fragments. By default, the visualization shows secondary structures predicted with P_a_-P_i_ > 0.7. In addition to visual representations, users can download the prediction results in various formats, such as Excel or PDF, for further analysis and integration into their research.

**Figure 3:**
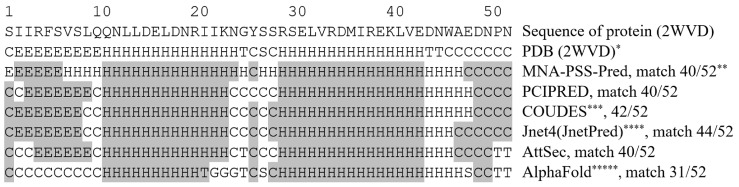
Comparison of SSP prediction results for different methods for the part of the sequence of b1 Putative nickel-responsive regulator protein of Helicobacter pylori (UniProt ID O25896) from PDB 2WVD record (52 amino acid residues) obtained from the ASTRAL dataset. The gray color reflects the correct prediction. * PDB (2WVD)—the appropriate data of SSP were extracted from PDB record 2WVD. ** match 40/52 means that the prediction of SSP was made correctly for 40 from 52 amino acid residues. *** COUDES is a combined method based on PSIPRED and an additional model of estimation of the presence and the type of β-turns, using a straightforward approach based on PSI-BLAST propensities and multiple alignments. **** Jnet4 (JnetPred) is a consensus model based on prediction results of different neural networks models based on PSSM (position-specific substitution matrix) and HHM (hidden Markov models) approaches. ***** The prediction results for AlphaFold were given from the AF-A0A0M8NUM1-F1-v4 record.

The comparison of prediction results made by the developed method (MNA-PSS-Pred) and other methods is represented in Figure 3. The part of the sequence of b1 Putative nickel-responsive regulator protein of Helicobacter pylori (UniProt ID O25896) from PDB 2WVD record (52 amino acid residues) was obtained from the ASTRAL dataset. This PDB record was not used for the training of MNA-PSS-Pred. The match shows the number of positions with correctly predicted SSPs from 52 amino acid residues. MNA-PSS-Pred, PCIPRED, and AttSec have equal accuracy. COUDES and Jnet4 showed the best accuracy in this example, but they are consensus models. AlphaFold showed less accurate prediction results. The results of prediction by different methods are the same in many positions, but in some positions, each method gives more accurate results.

The web application also provides detailed prediction information, including the positions of each predicted structure within the sequence, the specific subsequences, and the type of predicted SSP. The probability scores (P_a_-P_i_) for each position are also displayed, reflecting the confidence level of the predictions and continuous sequences of at least four amino acids. The buttons Copy, Excel, and PDF allow saving the data from the table with the predicted results. The search field helps to select rows in the table with the appropriate text, for example the sequence of protein fragment or type of SSP.

Using Peptide mode, one can make predictions for protein fragments and estimate their SSP types. This may be helpful because peptide sequences may belong to several SSP types in different proteins (see the table in Material and Methods).

Another direction for Peptide mode application is the analysis of the relation between amino acid substitutions and changing the type of SSP. Such analysis can help assess the impact of a genetic variant on changes in protein conformation. That is one of the tasks of clinical bioinformatics and medicine genetics during the annotation of genetic variants.

The prediction of secondary structures of antimicrobial peptides is used for drug development [23]. Peptide mode may be used for such a purpose. Antimicrobial peptides are classified according to their secondary structure, and this may be related with their mechanism of action. The presence of a secondary structure of antimicrobial peptides during their modification is important for their activity. The following types are distinguished: α-helical, β-sheet, mixed (α-helical/β-sheet), and cyclic structures, as well as those with no pronounced secondary structure in solutions. We made predictions for some well-known antimicrobial peptides using Peptide mode (Table 3).

Table 3 shows that the appropriate SSP types were correctly predicted for α-helical and β-sheet antimicrobial peptides. The experimental data in the PDB records also confirmed the SSP types of these antimicrobial peptides. The value of the prediction result for Fowlicidin-3 being less than for Magainin and Cathelicidin may be explained by there being only 10 amino acid residues of 27 (37%) belonging to α-helix according to 2HFR, while magainin includes 11 amino acid residues of 23 (48%) belonging to α-helix (2LSA). Cathelicidin includes 31 of 37 amino acid residues (84%) belonging to α-helix (5NMN). Protegrin-4 and Protegrin-5 include 10 of 18 amino acid residues (56%) belonging to β-sheet according to 6QKF and 2NC7, respectively.

## 3. Discussion

In this study, we aimed to enhance the prediction accuracy of SSP by developing SSPR models that utilize MNA descriptors for molecular fragments of proteins. Through the MultiPASS software, we developed SSPR models that have demonstrated a high level of accuracy, with an average prediction accuracy (AUC) of 0.902 for eight types of SSP. The created SSPR model was validated using the ASTRAL and CB513 datasets, achieving 0.860 and 0.889 AUC values in peptide mode, Q8 accuracies of 70.92% and 74.74%, respectively, and Q3 accuracies of 77.32% and 78.78%, respectively. These results indicate that while our model shows a competitive performance, there is still room for improvement when compared to other state-of-the-art models such as SSREDN, CRRNN, WGACSTCN, and AttSec. These comparisons underscore the need for a further refinement of our approach to achieve a higher prediction accuracy. 

There also exist AlphaFold and several other associated software predictors of protein structure (e.g., ESMFold or RosettaFold), which shown great performance. However, these models primarily focus on predicting the tertiary structure of proteins, operating by directly predicting three-dimensional coordinates. In contrast, our study is centered on predicting the secondary structure of proteins. Considering these differences, we did not perform a direct comparison between our models and those like AlphaFold. These models do not have a separate version specifically designed for the prediction of secondary structure of proteins. Instead, we compared the proposed method to other recently developed approaches specialized in secondary structure prediction.

Along with computer methods for predicting the secondary structure of proteins, there has been progress in the creation of experimental methods for their assessment. They are becoming more accessible, faster, and cheaper. Examples of such methods are a single unassigned 1D 13C nuclear magnetic resonance spectrum (1D ^13^C NMR spectrum) [24], Fourier transform IR spectroscopy [25], and circular dichroism [26]. Despite the development of these technologies, not everyone can use them in their research.

Our approach has a primary limitation that stems from the composition of the training set consisting of peptide sequences with a minimum length of four amino acids. This constraint means the model cannot firmly predict SSP for segments shorter than four amino acids. Such short sequences often play important roles in protein function and dynamics, and their exclusion from the accurate prediction limits the model’s applicability in understanding complete protein structures. This decision has been made because very short sequences, such as single amino acids, can represent a variety of SSP-like turns or coils, potentially leading to overfitting or insufficient learning by the model.

Analysis of SSP sequences revealed several features that may affect the accuracy of prediction. Short SSP sequences of four amino acid residues are less specific and often belong to two or more SSP types. Prediction results for such sequences also include multiple SSP types, some of which are false positives. There are also cases involving protein PDB entries with slightly different 3D structures between the individual protein and the protein interacting with other macromolecules or compounds. Such interactions may result in changes in SSP types for the same sequence.

Another significant challenge is the model’s method of generating and selecting subsequences from full-length proteins. The web application generates every possible subsequence within the specified length range from the input of an amino acid sequence and then predicts each subsequence’s structure. The predictions come with likelihood scores (P_a_-P_i_), and subsequently, the highest scores are selected. However, a high-likelihood score does not guarantee that the selected subsequences will accurately represent the overall secondary structure of the full-length protein.

The freely available MNA-PSS-Pred web application can be useful in various studies related to the determination of SSP or their changes through amino acid substitutions, for example, epitope selection for vaccine development or identification of pathogenic or drug-resistant amino acid substitutions.

## 4. Materials and Methods

The input data consisted of entries chosen from the UniProt protein database [27], specifically from the ’Reviewed (Swiss-Prot) Proteins’ category. This category was selected because all proteins within it had undergone peer-reviewing, providing a dependable foundation for the dataset. mmCIF files [28] were acquired from the RCSB PDB 3D structures database (November 2023) [4]. Proteins were selected with a focus on 3D structures with a resolution of 2Å or less to ensure the high accuracy of secondary structure elements. The 3D structures were predominantly obtained using X-ray diffraction (335,500 SSP sequences), with the remaining structures derived from a combination of neutron diffraction, electron crystallography, electron paramagnetic resonance, and electron microscopy (800 SSP sequences). The amino acid sequences of all types of SSPs represented as peptides were annotated using DSSP 4.0.4 (available in September 2023) based on the records of 3D protein structures from PDB [5]. The training set included 336,275 records of SSPs (323,356 unique sequences) with lengths from 4 up to 130 amino acids from 37,600 unique proteins. Some SSP sequences simultaneously belonged to different types of secondary structures. Therefore, in terms of redundancy, the training dataset was unique on 96.2%. We also analyzed the number of cases where SSP sequences belonged to two or more SSP types. The number of such cases for some SSP types was significant (Table 4). There were 15620 such sequences in the data, most of which were in the length of four amino acids (14524 sequences, 0.93% of all those with two or more SSP types). These sequences often also included two or three overrepresented amino acid residues such as A, L, E, G, V, K, or S (e.g., AAAS belongs to G, H, T types of secondary structures; QLEE belongs to E, T, H types of secondary structures). This may affect the prediction accuracy of such sequences and types of SSP, leading to their overprediction. Nevertheless, in this study we decided to leave the peptide sequences belonging to different SSP types in the training set, as this reflected the real picture of the experimental data. The number of SSP sequences belonging to only one SSP type is underlined in bold in Table 4.

Table 4 shows that peptide sequences annotated as G, P, T, and S types of SSP can often belong to other SSP types. More than 12% of G sequences were also annotated as H. A significant part of P, T, and S sequences were also annotated as C (unstructured coils)—21.2%, 12.7%, and 15.6%, respectively.

**Figure 4:**
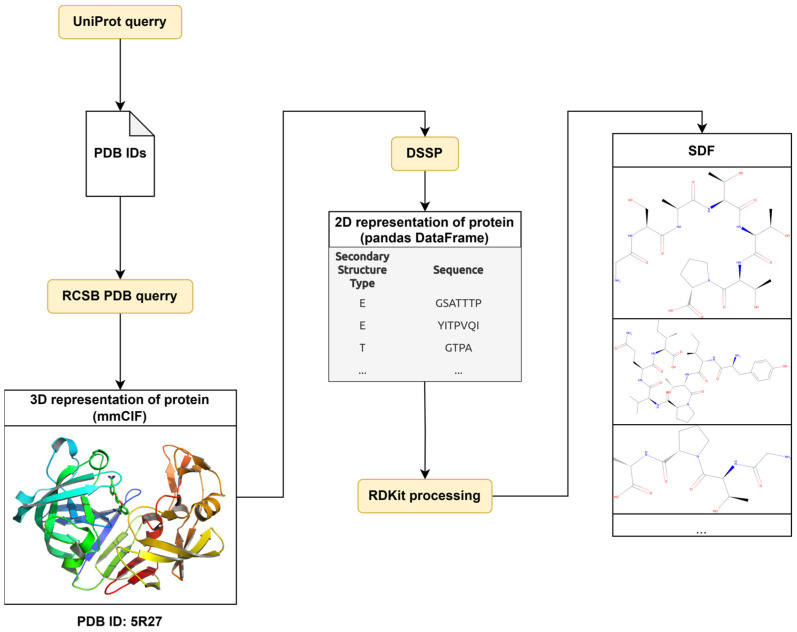
Preprocessing pipeline. The data on PDB records were extracted from PDB using UniProt data. The given experimentally determined 3D structures were analyzed by DSSP for annotation of SSP. The protein sequences were divided into peptide sequences according to their SSP annotation. The peptide sequences were converted by RDKit from one-letter code to structural formulae in SD file format.

The structural formulas of these peptide sequences were converted into SDF (Structure Data File, developed by MDL Information Systems) format and used as a training set for MultiPASS software (Figure 4).

**Figure 5:**
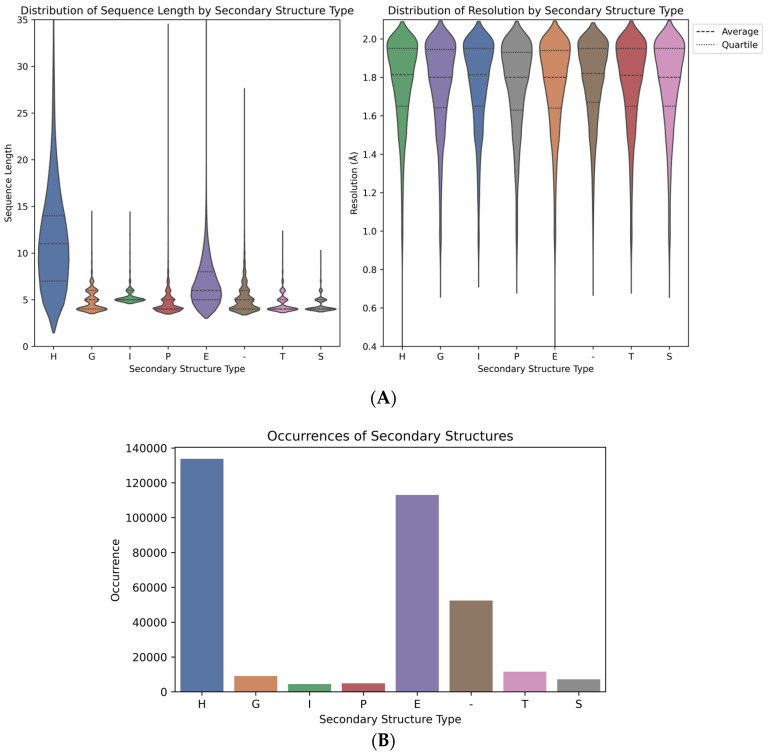
The secondary structures are classified as H (α-helix), G (3_10_-helix), I (π-helix), P (polyproline II helix), E (extended strand), - (unstructured coil, C), T (turn), and S (bend). (**A**) density of sequence lengths and resolution values for each secondary structure type, with averages and quartiles marked for clarity (**B**) frequency of each secondary structure type.

The data from the training set are represented in a CSV file in the Supplementary Materials. The distribution of the resolution and sequence lengths of SSP sequences is represented in Figure 5.

The violin plots on Figure 5A illustrate the density of sequence lengths and resolution values for each secondary structure type, with averages and quartiles marked for clarity. Notably, the H class displays a broad distribution with a higher average sequence length compared to other structures and can spread up to 130 amino acids. The bar chart on Figure 5B indicates the frequency of each secondary structure type, with H and E classes being the most prevalent. The B class (β-bridge) was not included in the further study due to its limited prevalence and relatively short sequences.

In this study, the ASTRAL [29] and CB513 [30] datasets were employed to benchmark the performance of the created SSPR models.

**Figure 6:**
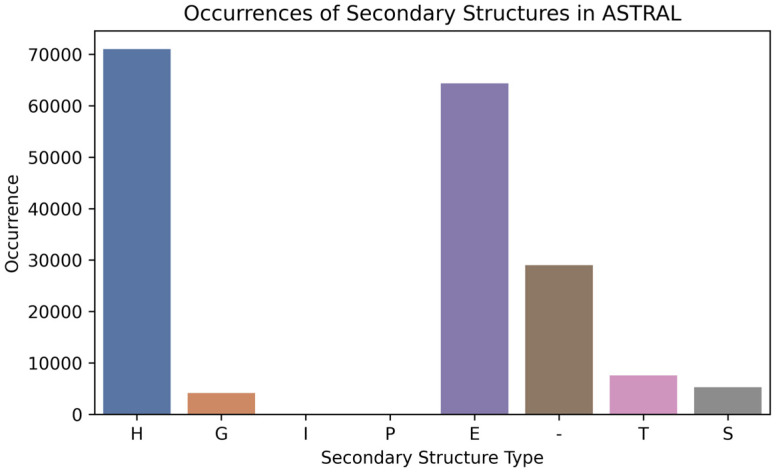
Occurrences of secondary structures in ASTRAL: H (α-helix), G (3_10_-helix), I (π-helix), P (polyproline II helix), E (extended strand), - (unstructured coil, C), T (turn), and S (bend).

The ASTRAL dataset is derived from the SCOPe (Structural Classification of Proteins —extended) database, which provides a detailed and comprehensive classification of protein structures based on their evolutionary and structural relationships. ASTRAL offers a curated subset of protein domains from the PDB database with high-quality structural annotations, facilitating accurate and efficient comparisons of protein structures. The dataset is designed to reduce redundancy and ensure that the included protein domains represent a wide range of structural families without significant sequence similarity. Based on the ASTRAL dataset, we created a test set from 181,429 unique peptide sequences of SSPs with a length of four and more amino acid residues. There was an overlap of 73,993 SSPs between the original training set and the ASTRAL dataset, which was 22.88% of the training set and 40.77% of the ASTRAL set. All ASTRAL SSP sequences were removed from the original training set during validation. The distribution of SSP sequences according to their types is represented in Figure 6.

**Figure 7:**
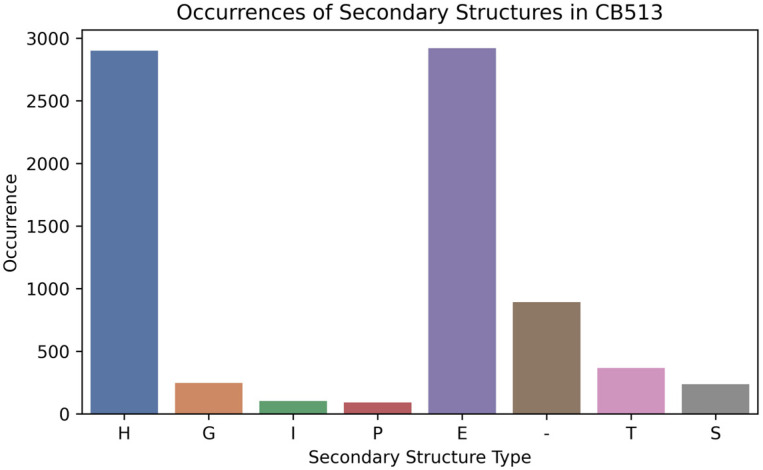
Occurrences of secondary structures in CB513: H (α-helix), G (3_10_-helix), I (π-helix), P (polyproline II helix), E (extended strand), - (unstructured coil, C), T (turn), and S (bend).

The CB513 dataset is a benchmark set comprising 513 non-redundant protein sequences with known secondary structures, sourced from the Protein Data Bank (PDB). The sequences in CB513 vary widely in length, complexity, and structural motifs, providing a comprehensive test for assessing the accuracy and robustness of SSP prediction algorithms. Only SSP peptides with a length of four and more amino acid residues were used. The CB513 test set, including 7760 peptide sequences of SSPs, was created based on the CB513 dataset (Figure 7). There was an overlap between the initial training set and CB513 datasets (5908 SSP sequences), which was 1.8% of the training set and 76.9% of the CB513 set. All overlapping CB513 SSP sequences were removed from the original training set during validation. There was also an overlap between the ASTRAL and CB513 datasets of 3886 SSP sequences, which was 2.1% of the ASTRAL set and 50.6% of the CB513 set. 

The data on SSP peptides from the CB513 and ASTRAL test sets are represented in CSV files in the Supplementary Materials.

The data on structural formulae of peptides in MDL MOL v3000 format and their belonging to the type of SSPs were kept in SD (Structure-Data) files (SDF) [31]. MDL MOL v3000 format allows writing the structural formula of a macromolecule with a large number of atoms in the structure. In this study, we used multilevel neighborhoods of atoms (MNA) descriptors for the representation of structural formulae of peptides while creating SSPR models [32]. To generate MNA descriptors, only the names of atoms and a list of bonds from the generated SD files are used.

MultiPASS software was used to create SSPR models [17,18,19,20,21,22]. MultiPASS is a special version of PASS (Prediction of Activity Spectra for Substances) software [33], which has been used for over 30 years to predict biological activities for low-weight organic molecules. A special feature of MultiPASS is the utilization of varying levels (the distance in bonds from the central atom) of MNA descriptors, up to 30, to describe structural formulae of peptides.

MNA descriptors represent the structure of a molecule in the form of a set of strings of atom-centered fragments linear notation. MNA descriptors are generated iteratively, beginning with a basic description of each atom and progressively incorporating descriptions of neighboring atoms. In chemoinformatics, it is considered that structural formulae represent properties of molecules. We believe this is also true for relationships between structural formulae of peptides and SSP types, which may be considered as their property.

In MultiPASS, after the generation of MNA descriptors, a Bayesian classifier is used to identify relationships between MNA descriptors and the type of SSPs. For each SSP type, positive examples were peptide structures annotated with the corresponding SSP type, whereas negative examples were all other SSP sequences not belonging to this SSP type.

The result of MultiPASS prediction includes P_a_ and P_i_ values for the predicted type of SSP. P_a_ (probability “to be active”) estimates the chance that the studied peptide belongs to the type of the appropriate SSP (resembles the structures of peptides that are the most typical in a sub-set of “actives” in the MultiPASS training set). We may consider the relation of the appropriate peptide with the class of SSP as its property (“activity”). P_i_ (probability “to be inactive”) estimates the chance that the studied peptide does not belong to the type of the appropriate SSP (resembles the structures of peptides that are the most typical in the sub-set of “inactives” in the MultiPASS training set). Only SSPs with P_a_ > P_i_ are considered as possible for a particular peptide. The highest P_a_ > P_i_ value means the highest probability to confirm the predicted result in the experimental study. MultiPASS also includes the leave-one-out cross-validation procedure, which during the training of SSPR model calculates an estimation of accuracy, the invariant accuracy of prediction (IAP). IAP is an assessment of the probability that positive and negative examples, randomly selected from the test set, can be correctly classified by a model. IAP is numerically equal to the ROC AUC value traditionally used to assess the accuracy of classification models.

In this study, the ASTRAL and CB513 datasets were employed to benchmark the performance of the created SSPR model on whole-length sequences of proteins. The primary metrics used to evaluate the performance of the models are the Q3 and Q8 accuracies that are defined as:

(1) $$Q=100\%\cdot \frac{1}{N}\sum_{i=1}^{N}\delta \left(y_{i},\hat{y_{i}}\right),$$

where $\delta \left(y_{i},\hat{y_{i}}\right)$ is defined as:

(2) $$\delta \left(y_{i},\hat{y_{i}}\right)=\left\{\begin{array}{cc} 1 & \mathrm{if}\;y_{i}=\hat{y_{i}}\\ 0 & \mathrm{if}\;y_{i}\neq \hat{y_{i}} \end{array}\right.$$

where $y_{i}$ and $\hat{y_{i}}$ are the true and predicted secondary structure for the i-th residue, and N is the total number of residues in the dataset. 

Q3 and Q8 are measures of prediction accuracy for three (H, E, C) and eight (H (α-helix), G (3_10_-helix), I (π-helix), P (polyproline II-helix), E (extended strand), C (unstructured coil), T (turn), and S (bend)) types of SSP, respectively.

To predict that a residue belongs to a particular type using SSPR models, the following algorithm was used. All possible subsequences from 4 to 30 amino acids in length were generated by sliding the window for a protein sequence. Then, these subsequences were converted to an SD file used as input for MultiPASS with the loaded SSPR model. Based on the prediction results, each position of a residue in a protein was annotated by the maximal predicted P_a_-P_i_ value among all subsequences.

AUC value was used as one of characteristic of accuracy of SSP prediction. The AUC is the area under the ROC (receiver operating characteristic) curve. For each class, a separate ROC is created by treating that class as the positive class and the rest as the negative class. Then, the AUC is calculated for each class, and an average AUC score is obtained to evaluate overall model performance.

## Figures and Tables

**Table 1:** Characteristics of training sets and accuracy of prediction (AUC_LOO CV_) for the SSPR model created by 9th level of MNA descriptors before (initial model) and after excluding ASTRAL and CB513 data.

SSP	Description	Initial Model		Model Without ASTRAL Data		Model Without CB513 Data	
		N	AUC_LOO CV_	N	AUC_LOO CV_	N	AUC_LOO CV_
H	α-helix	134,847	0.925	107167	0.929	132571	0.925
G	3_10_-helix	10,595	0.884	6779	0.876	10269	0.883
I	π-helix	4410	0.876	4309	0.883	4337	0.876
P	Polyproline II-helix	5436	0.927	3690	0.928	5304	0.927
E	β-strand	113,175	0.900	81693	0.899	110665	0.900
C	Unstructured coil	52,466	0.888	40543	0.892	51516	0.888
T	Turn	13,349	0.905	8072	0.898	12911	0.904
S	Bend	8167	0.914	4915	0.913	7894	0.914

G, I, and C classes provided a less accurate prediction (AUC_LOO CV_ is less 0.9) in comparison to other types of SSP (AUC_LOO CV_ is 0.9 or higher). For 3_10_-helix and π-helix, it may be explained by the small size of sequences, part of which may be close to other types of SSP. The large structural diversity may explain the lower accuracy of prediction for the C class of SSP. In general, the created SSPR models displayed a high level of accuracy (mean AUC_LOO CV_ was 0.902). They were implemented in a freely available web application (MNA-PSS-Pred).

**Table 2:** Accuracy of prediction calculated for ASTRAL and CB513 test sets.

SSP	TP	FP	TN	FN	Sensitivity	Specificity	Accuracy	Balanced Accuracy	AUC
*ASTRAL test set*									
H	58,431	17,677	92,763	12,557	0.823	0.840	0.833	0.832	0.907
G	3179	39,531	137,749	969	0.766	0.777	0.777	0.772	0.853
I	60	41,020	140,310	38	0.612	0.774	0.774	0.693	0.758
P	-	-	-	-	-	-	-	-	-
E	51,903	21,977	95,109	12,439	0.807	0.812	0.810	0.810	0.885
C	21,992	34,482	117,953	7001	0.759	0.770	0.771	0.767	0.849
T	5926	33,107	140,741	1654	0.782	0.810	0.808	0.796	0.870
S	4306	31,863	144,287	972	0.816	0.819	0.819	0.818	0.899
mean					0.770	0.800	0.799	0.784	0.860
*CB513 test set*									
H	2417	632	4228	482	0.834	0.870	0.856	0.852	0.924
G	203	1797	5714	45	0.818	0.761	0.763	0.790	0.867
I	79	1822	5834	24	0.767	0.762	0.762	0.765	0.836
P	82	1302	6366	9	0.901	0.830	0.831	0.866	0.935
E	2462	952	3886	459	0.843	0.803	0.818	0.823	0.894
C	724	1812	5055	168	0.812	0.736	0.745	0.774	0.850
T	318	1533	5859	49	0.866	0.792	0.796	0.829	0.905
S	205	1443	6078	33	0.861	0.808	0.810	0.835	0.899
mean					0.838	0.795	0.798	0.817	0.889

H (α-helix), G (3_10_-helix), I (π-helix), P (polyproline II helix), E (extended strand), C (unstructured coil), T (turn), and S (bend).

**Table 3:** Prediction results of SSP for antimicrobial peptides.

Name	Organism	PDB ID	Sequence	Pa-Pi	SSP
*α-helical*					
Magainin	Xenopus laevis	2LSA	GIGKFLHSAKKFGKAFVGEIMNS	0.672	Alpha helix
Cathelicidin	Homo sapiens	5NMN	LLGDFFRKSKEKIGKE FKRIVQRIKDFLRNLVPRTES	0.688	Alpha helix
Fowlicidin-3	Gallus gallus	2HFR	KRFWPLVPVAINTVAAGINLYKAIRRK	0.195	Alpha helix
*β-sheet*					
Protegrin-4	Sus scrofa	6QKF	RGGRLCYCRGWICFCVGR	0.479	Strand
Protegrin-5	Sus scrofa	2NC7	RGGRLCYCRPRFCVCVGR	0.240	Strand

Name—the name of peptide. SSP—the predicted type of SSP.

**Table 4:** The number of unique peptide sequences belonging to SSP types.

SSP	H	G	I	P	E	C	T	S
*Number of SSP sequences*								
H	**131,611**	1594	51	342	1677	2203	1338	522
G	1594	**7742**	4	200	747	1230	981	335
I	51	4	**4342**	0	56	19	11	1
P	342	200	0	**4393**	386	1560	259	213
E	1677	747	56	386	**111,144**	3368	888	525
C	2203	1230	19	1560	3368	**43,012**	2098	1614
T	1338	981	11	259	888	2098	**10,010**	895
S	522	335	1	213	525	1614	895	**6232**
Total	139,338	12,833	4484	7353	118,791	55,104	16,480	10,337
*Percentage of SSP sequences*								
H	**94.45**	12.42	1.14	4.65	1.41	4.00	8.12	5.05
G	1.14	**60.33**	0.09	2.72	0.63	2.23	5.95	3.24
I	0.04	0.03	**96.83**	0.00	0.05	0.03	0.07	0.01
P	0.25	1.56	0.00	**59.74**	0.32	2.83	1.57	2.06
E	1.20	5.82	1.25	5.25	**93.56**	6.11	5.39	5.08
C	1.58	9.58	0.42	21.22	2.84	**78.06**	12.73	15.61
T	0.96	7.64	0.25	3.52	0.75	3.81	**60.74**	8.66
S	0.37	2.61	0.02	2.90	0.44	2.93	5.43	**60.29**
Total	100.0	100.0	100.0	100.0	100.0	100.0	100.0	100.0

H (α-helix), G (3_10_-helix), I (π-helix), P (polyproline II helix), E (extended strand), C (unstructured coil), T (turn), and S (bend).

## Data Availability

The original data presented in the study are openly available in the MNA-PSS-Pred web application at https://www.way2drug.com/MNA2DFinder/definition.php.

## Supplementary Materials

The supporting information can be downloaded at: https://www.mdpi.com/article/10.3390/ijms252312525/s1.
